# COVID-19, maternal, and neonatal outcomes: National Mother-Child Cohort (NMCC) of K-COV-N cohort in South Korea

**DOI:** 10.1371/journal.pone.0284779

**Published:** 2023-04-20

**Authors:** Jongmin Oh, Whanhee Lee, Choong-jong Kim, Yi Jun Kim, Hyesook Park, Ji Hyen Lee, Mi Hye Park, Seulbi Lee, Eunhee Ha, Kyung A. Lee

**Affiliations:** 1 Department of Environmental Medicine, College of Medicine, Ewha Womans University, Seoul, Republic of Korea; 2 Institute of Ewha-SCL for Environmental Health (IESEH), College of Medicine, Ewha Womans University, Seoul, Republic of Korea; 3 School of Biomedical Convergence Engineering, College of Information and Biomedical Engineering, Pusan National University, Yangsan, Republic of Korea; 4 Department of Internal Medicine, Division of Infectious Diseases, Ewha Womans University School of Medicine, Seoul, South Korea; 5 Department of Preventive Medicine, Ewha Womans University College of Medicine, Seoul, Korea; 6 Graduate Program in System Health Science and Engineering, College of Medicine, Ewha Womans University, Seoul, Republic of Korea; 7 Department of Pediatrics, Ewha Womans University College of Medicine, Seoul, Republic of Korea; 8 Department of Obstetrics and Gynecology, Ewha Womans University College of Medicine, Seoul, Republic of Korea; 9 Department of Big Data Strategy, National Health Insurance Service, Wonju, Korea; Shahid Beheshti University of Medical Sciences, ISLAMIC REPUBLIC OF IRAN

## Abstract

Throughout the COVID-19 pandemic, pregnant women have been classified as a vulnerable population. However, the evidence on the effect of infection during pregnancy on maternal and neonatal outcomes is still uncertain, and related research comprising a large population of pregnant women in Asian countries is limited. We constructed a national cohort including mothers and children (369,887 pairs) registered in the Prevention Agency-COVID-19-National Health Insurance Service (COV-N), from January 1, 2020 to March 31, 2022. We performed propensity score matchings and generalized estimation equation models to estimate the effect of COVID-19 on maternal and neonatal outcomes. In summary, we found little evidence of the effect of COVID-19 infection during pregnancy on maternal and neonatal outcomes; however, a relationship between COVID-19 infection in the second trimester and postpartum hemorrhages was discovered (Odds ratio (OR) of Delta period: 2.26, 95% Confidence intervals (CI): 1.26, 4.05). In addition, neonatal intensive care unit (NICU) admissions increased due to COVID-19 infection (pre-Delta period: 2.31, 95% CI: 1.31, 4.10; Delta period: 1.99, 95% CI: 1.47, 2.69; Omicron period: 2.36, 95% CI: 1.75, 3.18). Based on the national retrospective cohort study data, this study investigated the effects of COVID-19 infection on maternal and neonatal outcomes in Korea from the pre-Delta to the initial Omicron epidemic periods. Our evidence suggests that the timely and successful policies of the government and academia in response to COVID-19 infections in newborns in Korea may cause an increase in NICU admissions, but nonetheless, they prevent adverse maternal and neonatal outcomes simultaneously.

## Introduction

Coronavirus disease 19 (COVID-19) infection has affected public health worldwide, and the pattern of coronavirus variant types have changed rapidly. In South Korea, three epidemics of great magnitude of COVID-19 infection (pre-Delta, Delta, and Omicron periods) were experienced from 2020 to early 2022. Although evidence on the Omicron variant suggests less virulence compared with the Delta strain, it has a greater immune vaccine escape. [1, 2]. The characteristics of the three variants (pre-Delta, Delta, and Omicron) are different, and there is a paucity of information on large study population maternal and neonatal outcomes during each variant period.

Since early 2020, the COVID-19 pandemic has resulted in tremendous public health concerns. In particular, pregnant women have been classified as a vulnerable population to COVID-19 infection from the very start of the pandemic. However, the epidemiological evidence for infection risk during pregnancy on maternal and neonatal outcomes, such as preterm birth, hypertensive disorders in pregnancy, and postpartum hemorrhage of mothers and prematurity, respiratory and neurodevelopmental disorders of neonates, is still undetermined. Firstly, several previous studies were conducted at selected medical institutions, thus creating a risk of selection bias [1, 3, 4]. Secondly, compared to non-Western countries, many studies have been performed in Western countries where health outcomes, quarantine policies, lifestyles, and living environments related to COVID-19 are heterogeneous [1, 5]. Lastly, most of the existing studies examined the association between viral infection and maternal or neonatal outcomes; however, information on the casual relationship between COVID-19 and maternal-neonatal outcomes via advanced analytic approaches is limited [3, 6, 7].

We constructed a national mother-child cohort (NMCC) from January 1, 2020 through March 31, 2022 from the Korea Disease Control and Prevention Agency-COVID19-National Health Insurance Service cohort (K-COV-N cohort), and conducted a thorough analysis to explore our hypothesis that a causal association between COVID-19 infection and maternal and neonatal outcomes exists.

## Materials and methods

### Study population

**Fig 1:**
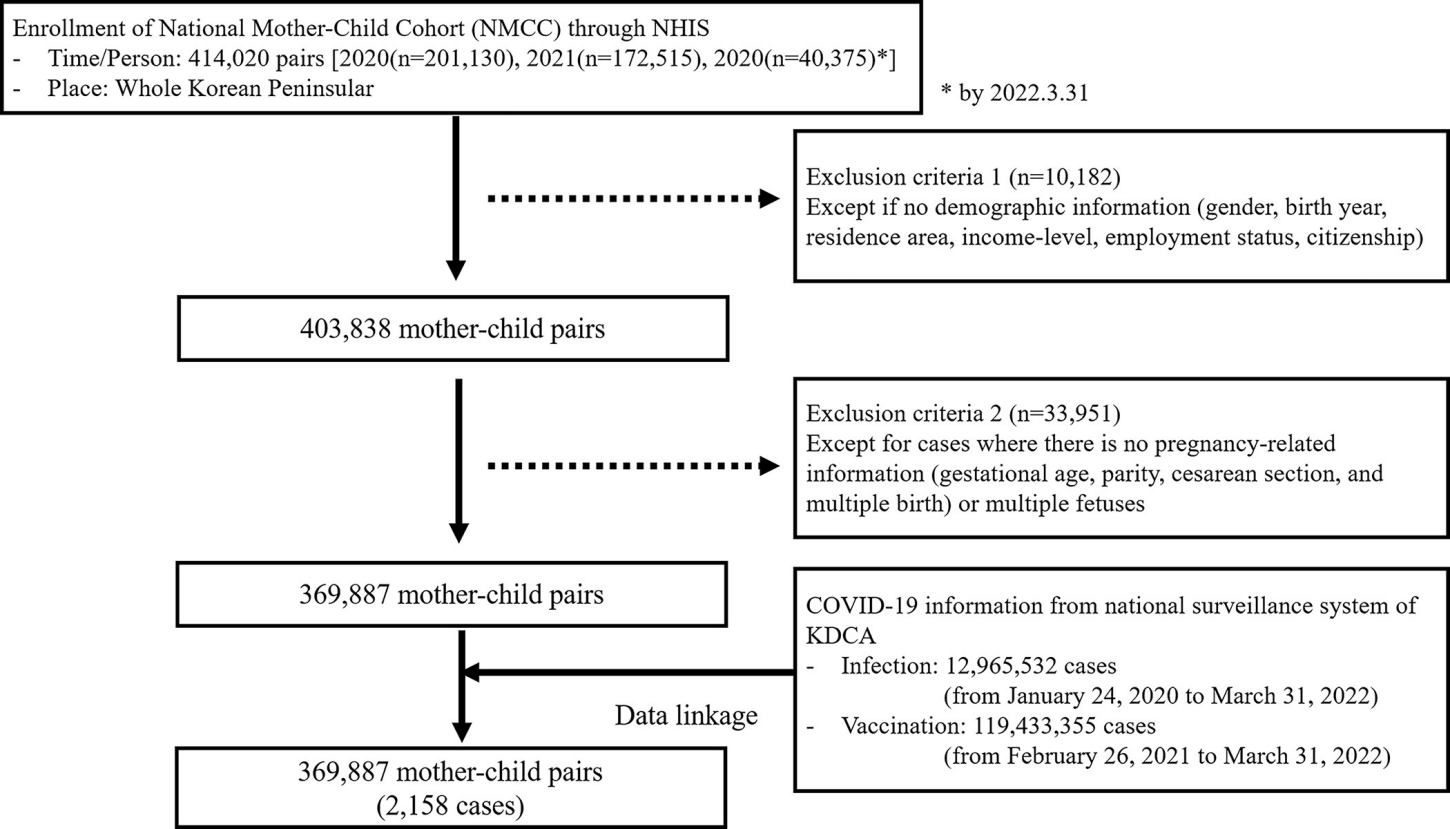
Enrollment of National Mother-Child Cohort (NMCC) for the association of COVID-19 infection during pregnancy with maternal and neonatal outcomes.

We obtained the data of 414,020 mothers and children as a cohort from the mother-child pair database in K-COV-N cohort of the National Health Insurance Service (NHIS) from January 1, 2020 to March 31, 2022 to examine the relationship between maternal and newborn outcomes of COVID-19 infection (Fig 1). Participants were defined by applying two exclusion criteria. First, we excluded participants without demographic information (sex, age, income-level, employment status, residence area, and citizenship). Second, participants were excluded if there was no pregnancy-related information (gestational age, date of delivery, and parity) or if there were multiple fetuses. Therefore, a total of 369,887 mother-child pairs were defined as the subjects of this study.

### Ethical approvals

This study was exempted from review by the Institutional Review Board of Ewha Womans University Seoul Hospital (IRB No: 2022-05-038).

### Cohort database

The NMCC is a mother-child pair cohort from the K-COV-N cohort in the NHIS of South Korea and is linked to COVID-19 confirmed case surveillance data operated by the Korea Disease Control and Prevention Agency (KDCA) from January 21, 2020 through March 31, 2022. The NHIS is a mandatory system for people who were born in South Korea, thus the NHIS data theoretically covers the entirety of mothers and newborns living in South Korea, except for, temporary foreign residents and Korean citizens who have resided overseas long term [8]. The NHIS data contain information on claims, pregnancy-delivery databases, and mother-child linkages. In addition, the confirmed case surveillance data provided by the KDCA include information on COVID-19 confirmed cases and the vaccination status of all residents in South Korea. The NHIS and the KDCA data were linked by de-identified registration numbers.

The mothers were defined as females who were registered in the mother-child pair database in the NHIS with at least one delivery record during the study period. We excluded mothers without medical records, demographic information, or pregnancy-related variables and mothers with multiple births (Fig 1). We also investigated the cases related to initial infection. Based on the KDCA data, case study groups were defined as mothers who were confirmed COVID-19 positive by polymerase chain reaction (PCR) diagnostic tests during pregnancy. Mothers who did not have confirmation records were used as a control group.

### Maternal and neonatal outcomes

A total of six outcomes were addressed in this study: (1) maternal and neonatal outcomes, (2) maternal outcomes, (3) neonatal outcomes, (4) preterm birth (PTB; less than 37 weeks of pregnancy), (5) postpartum hemorrhages (PPH), and (6) admission to the neonatal intensive care unit (NICU) that was not a direct cause of COVID-19 infection. All outcome variables were binary variables, i.e., “Yes” or “No”. Detailed information on these outcomes is reported in the S1 Text and S1 Table.

### Sub-period analysis

To investigate the heterogeneous effects of COVID-19 infection over time, we divided the study period into three sub-periods, which are closely related to the variant types that were dominant during each sub-period: the pre-Delta (January 2020—June 2021), the Delta (July 2021—December 2021), and the Omicron (January 2022—March 2022) periods. In addition, we selected the control groups corresponding to the sub-periods as non-infectious females who were pregnant during each sub-period.

### Covariates

To carry out a thorough analysis, the following covariates had to be considered. These factors included maternal age, gestational age, sex of child, income, employment status, residence area, citizenship, parity, cesarean section, underlying disease, and vaccination status. Income was classified into six categories (low, medium, and high groups based on the tertiles for each employee-type and for local-type beneficiaries). Beneficiaries who were affiliated with the company that supported their insurance were referred to as employee-type beneficiaries. Beneficiaries who did not fit into the employee type category were referred to as local-type beneficiaries. Employment status was defined by the Occupational Regulations (INDTP_CD) codes in the qualification database of the NHIS [9]. If any occupational information was identified, the subject was classified as a “worker” and if not, a “non-worker”.

During our investigation, we also assessed three underlying diseases as a variable using ICD-10 codes. These included cardiovascular disease (CVD), respiratory diseases, and diabetes. For CVD and respiratory diseases, we assigned the underlying disease variable to a subject if there were at least two hospital visits with ICD-10 diagnostic codes for CVD (O10, O16, I10-I15, and Q20-Q28) and respiratory diseases (A15-A19, J42-J45) within a year prior to pregnancy. Similarly, for diabetes, underlying disease was defined by at least two hospital visits with ICD-10 diagnostic codes for diabetes (E10-E14, O240-O244, and O249) within a year prior to pregnancy or before COVID-19 infection during the pregnancy.

Vaccination status was also considered in the model. The vaccination status of a subject was provided by KDCA data from February 26, 2021. To define the “complete vaccination” status, we considered the types of vaccine and the number of vaccinations the subject had. In Korea, a total of five types of COVID-19 vaccines were provided during the study period: Astrazeneca, Pfizer-BioNTech, Moderna, Janssen, and Novavax. Except for Janssen, we defined a mother to possess a complete vaccination status after the second vaccination. In the case of the COVID-19 group, a fully vaccinated mother was defined by a second vaccination undertaken prior to infection [10, 11].

### Statistical analysis

To address heterogeneous distribution of covariates (i.e., to find a causal association), we conducted matching methods based on a propensity score (PS). The propensity score was calculated using a logistic regression model including all the covariates previously stated, and the 1:4 greedy nearest neighbor matching technique (caliper = 0.2) without replacement. This was performed to estimate an average treatment effect (in our case, COVID-19 infection). We calculated standardized mean differences (SMD) for distribution of covariates before and after matching between groups (Table 1 and S1–S3 Figs) to establish the matching performance. From the matched samples, we estimated the odds ratio (ORs) and 95% confidence intervals (CIs) to determine the effect of COVID-19 on outcomes using the generalized estimation equation with the “*exchangeable*” correlation matrix.

**Table 1:** Baseline characteristics for pregnant women according to COVID-19 variant types before and after propensity score matching.

Time of the virus epidemic	pre-Delta period								Delta-period						Omicron period					
	All participants, n = 306,962					PS-matched participants, n = 1,330			All participants, n = 30,968			PS-matched participants, n = 3,315			All participants, n = 31,957			PS-matched, participants, n = 6,037		
**Characteristics**	Non-COVID-19		COVID-19		SMD	Non-COVID-19	COVID-19	SMD	Non-COVID-19	COVID-19	SMD	Non-COVID-19	COVID-19	SMD	Non-COVID-19	COVID-19	SMD	Non-COVID-19	COVID-19	SMD
**n**	306,696		266			1,064	266		30,305	663		2,652	663		30,728	1,229		4,809	1,228	
**Continuous variables (Mean ± SD)**																				
Maternal age	32.3 (4.5)	33.1 (4.6)		0.18		32.9 (4.5)	33.1 (4.6)	0.05	33.1 (4.4)	32.9 (4.3)	0.04	33.0 (4.4)	32.9 (4.3)	0.01	32.8 (4.3)	32.7 (4.3)	0.02	32.7 (4.3)	32.7 (4.3)	0.00
GA at delivery	38.8 (1.4)	38.8 (1.3)		0.01		38.8 (1.3)	38.8 (1.3)	0.02	38.5 (1.7)	38.7 (1.5)	0.08	38.7 (1.6)	38.7 (1.5)	0.01	38.9 (1.1)	39.2 (0.9)	0.30	39.2 (1.0)	39.2 (0.9)	0.00
**Categorical variables (No. (%))**																				
**Sex of child**																				
Boys	156,943 (51)	146 (55)		0.07		586 (55)	146 (55)	0.01	15,625 (52)	351 (53)	0.03	1,419 (54)	351 (53)	0.01	15,569 (51)	630 (51)	0.01	2,477 (52)	630 (51)	0.00
Girls	149,753 (49)	120 (45)		0.07		478 (45)	120 (45)	0.00	14,680 (48)	312 (47)	0.03	1,233 (46)	312 (47)	0.01	15,159 (49)	599 (49)	0.01	2,332 (48)	598 (49)	0.00
**Income-level**																				
Unknown	16,177 (5)	13 (5)		0.02		26 (2)	13 (5)	0.13	1,629 (10)	34 (5)	0.01	134 (5)	34 (5)	0.00	1,862 (6)	70 (6)	0.02	241 (5)	70 (6)	0.03
Local subscriber: low	30,429 (10)	30 (11)		0.04		100 (9)	30 (11)	0.06	3,463 (11)	98 (15)	0.10	373 (14)	98 (15)	0.02	2,861 (9)	138 (11)	0.06	549 (11)	138 (11)	0.01
Local subscriber: mid	30,138 (10)	24 (9)		0.03		112 (11)	24 (9)	0.05	2,919 (10)	73 (11)	0.05	288 (11)	73 (11)	0.00	3,003 (10)	123 (10)	0.01	469 (10)	123 (10)	0.01
Local subscriber: high	8,748 (3)	8 (3)		0.01		26 (2)	8 (3)	0.03	687 (2)	12 (2)	0.03	44 (2)	12 (2)	0.01	894 (3)	24 (2)	0.06	99 (2)	24 (2)	0.01
Employee subscriber: low	88,885 (29)	79 (30)		0.02		354 (33)	79 (30)	0.08	10,074 (33)	188 (28)	0.11	744 (28)	188 (28)	0.01	8,043 (26)	330 (27)	0.02	1,354 (28)	330 (27)	0.03
Employee subscriber: mid	102,720 (33)	84 (32)		0.04		343 (32)	84 (32)	0.01	9,259 (31)	191 (29)	0.04	795 (30)	191 (29)	0.03	10,537 (34)	410 (33)	0.02	1,605 (33)	409 (33)	0.00
Employee subscriber: high	29,599 (10)	28 (11)		0.03		103 (10)	28 (11)	0.03	2,274 (8)	67 (10)	0.09	274 (10)	67 (10)	0.01	3,528 (11)	134 (11)	0.02	492 (10)	134(11)	0.02
**Employment status**																				
Non-worker	75,460 (25)	66 (25)		0.00		260 (24)	66 (25)	0.10	7,621 (25)	195 (29)	0.10	750 (28)	195 (29)	0.02	7,249 (24)	274 (22)	0.03	1,042 (22)	274 (22)	0.02
worker	231,236 (75)	200 (75)		0.00		804 (76)	200 (75)	0.10	22,684 (75)	468 (71)	0.10	1,902 (72)	468 (71)	0.02	23,479 (76)	955 (78)	0.03	3,767 (78)	954 (78)	0.02
**Residence area**																				
Region 1	157,901 (51)	179 (67)		0.33		729 (69)	179 (67)	0.03	15,649 (52)	470 (71)	0.40	1,877 (71)	470 (71)	0.00	15,842 (52)	702 (57)	0.11	2,788 (58)	702 (57)	0.02
Region 2	72,926 (24)	51 (19)		0.11		191 (18)	51 (19)	0.03	7,092 (23)	105 (16)	0.19	439 (17)	105 (16)	0.02	7,237 (24)	291 (24)	0.00	1,119 (23)	291 (24)	0.01
Region 3	75,869 (25)	36 (14)		0.29		144 (14)	36 (14)	0.00	7,564 (25)	88 (13)	0.30	336 (13)	88 (13)	0.02	7,649 (25)	236 (19)	0.14	902 (19)	236 (19)	0.01
**Citizenship**																				
Domestic	286,884 (94)	247 (93)		0.03		990 (93)	247 (93)	0.01	28,420 (94)	600 (90)	0.12	2,400 (90)	600 (90)	0.00	29,186 (95)	1,173 (95)	0.02	4,603 (96)	1,172 (95)	0.01
Foreign	19,812 (6)	19 (7)		0.03		74 (7)	19 (7)	0.01	1,885 (6)	63 (10)	0.12	252 (10)	63 (10)	0.00	1,542 (5)	56 (5)	0.02	206 (4)	56 (5)	0.01
**Parity**																				
Primiparous women	177,537 (58)	144 (54)		0.08		533 (52)	144 (54)	0.04	17,976 (59)	352 (53)	0.13	1,460 (55)	352 (53)	0.04	18,369 (60)	623 (51)	0.18	2,478 (52)	623 (51)	0.02
Multiparous women	129,159 (42)	122 (42)		0.08		511 (48)	122 (46)	0.04	12,329 (41)	311 (47)	0.13	1,192 (45)	311 (47)	0.04	12,359 (40)	606 (49)	0.18	2,331 (48)	605 (49)	0.02
**Cesarean section**																				
No	137,818 (45)	117 (44)		0.02		480 (45)	117 (44)	0.02	12,333 (41)	255 (38)	0.05	1,005 (38)	255 (38)	0.01	12,073 (39)	486 (40)	0.01	1,838 (38)	485 (39)	0.03
Yes	168,878 (55)	149 (56)		0.02		584 (55)	149 (56)	0.02	17,972 (59)	408 (62)	0.05	1,647 (62)	408 (62)	0.01	18,655 (61)	743 (60)	0.01	2,971 (62)	743 (61)	0.03
**Underlying disease**																				
No	250,144 (82)	250 (94)		0.39		1,004 (94)	250 (94)	0.02	23,975 (79)	594 (90)	0.29	2,407 (91)	594 (90)	0.04	25,516 (83)	1,036 (84)	0.03	4,052 (84)	1,035 (84)	0.00
Yes	56,552 (18)	16 (6)		0.39		60 (6)	16 (6)	0.02	6,330 (21)	69 (10)	0.29	245 (9)	69 (10)	0.04	5,212 (17)	193 (16)	0.03	757 (16)	193 (16)	0.00
**Vaccination**																				
Not Complete	218,704 (71)	265 (100)		0.88		733 (69)	265 (100)	0.93	26,588 (88)	657 (99)	0.47	2,629 (99)	657 (99)	0.00	30,022 (98)	1,041 (85)	0.47	4,178 (87)	1,041 (85)	0.06
Complete	87,992 (29)	1 (0)		0.88		331 (31)	1 (0)	0.93	3,717 (12)	6 (1)	0.47	23 (1)	6 (1)	0.00	706 (2)	188 (15)	0.93	631 (13)	187 (15)	0.06

Abbreviations: SMD, absolute standardized mean difference; GA, gestational age.

Additionally, to examine whether the effects of COVID-19 infection differed between infection periods during pregnancy, we subdivided the outcomes by gestational trimesters: the confirmation in the first trimester (0 to 13 weeks), second trimester (14 to 27 weeks), and third trimester (28 weeks to the end of the pregnancy). An additional subgroup analyses was performed on age group (< 35 vs ≥ 35), employment status, parity, and vaccination status. Statistical analysis as described above was subsequently performed on all sub-groups and sub-periods.

### Sensitivity analysis

To investigate the robustness of our results, we performed a total of four sensitivity analyses. First, we applied different matching ratios (1:1). Then, we performed an analysis using a stabilized inverse propensity of treatment weighting to estimate the average treatment effect. In addition, we conducted an analysis exclusively on individuals of Korean nationality to examine the potential confounding effects of nationality. Finally, we also added body mass index (BMI) as a covariate using only mother beneficiaries who were eligible for BMI (n = 157,456; 1,095 cases of COVID-19) to examine the confounding effect of BMI. Information related to the BMI variable was collected from the regular health check-up database from the NHIS data within the two years prior to pregnancy. In our study data, BMI information was present only in subjects who had health checkups. Therefore, mothers without health check-up information were excluded.

All data processing and statistical analysis used SAS (version 9.4, SAS Institute Inc., Cary NC, USA) and R statistical software (version 4.0, R Foundation for Statistical Computing, Vienna, Austria). Data visualization used R and Geographic Information System (GIS) software ArcGIS 9.3 (ESRI, 2009). We used the “*dplyr*”, “*lubridate*”, “*ggplot2*”, “*MatchIt*” and “*optmatch*” packages in the R programming language.

## Results

A total of 369,887 mother-child pair datasets was included as study subjects. Among them were 2,158 (0.58%) cases of COVID-19 infection during pregnancy. This is broken down into 266 (0.07%) cases in the pre-Delta period, 663 (0.18%) cases in the Delta period, and 1,229 (0.33%) cases in the Omicron period (Table 1, S4 and S5 Figs).

In S2 Table, the average (±standard deviation) age of women that were pregnant was 32.4 (4.4) years and the average gestational age at delivery was found to be 38.8 (1.6) weeks. Of those pregnant women, 75% (n = 279,022) were employed and 25% (n = 90,865) were housewives. Furthermore, the cohort was comprised largely of domestic citizens, at 94% (n = 346,510) and foreign citizenship was 6% (n = 23,377). Parity was 58% (n = 215,001) for primiparous women and 42% (n = 154,886) for multiparous women and the rate of cesarean section procedures was 56% (n = 206,805). The percentage of pregnant women with an underlying disease was ~18% (n = 68,372). The vaccination status of the study groups was 75% (n = 277,277) for incomplete vaccination and 25% (n = 92,610) for complete vaccination. After PS matching, the balance between covariate groups was achieved (Table 1 and S1–S3 Figs).

**Fig 2:**
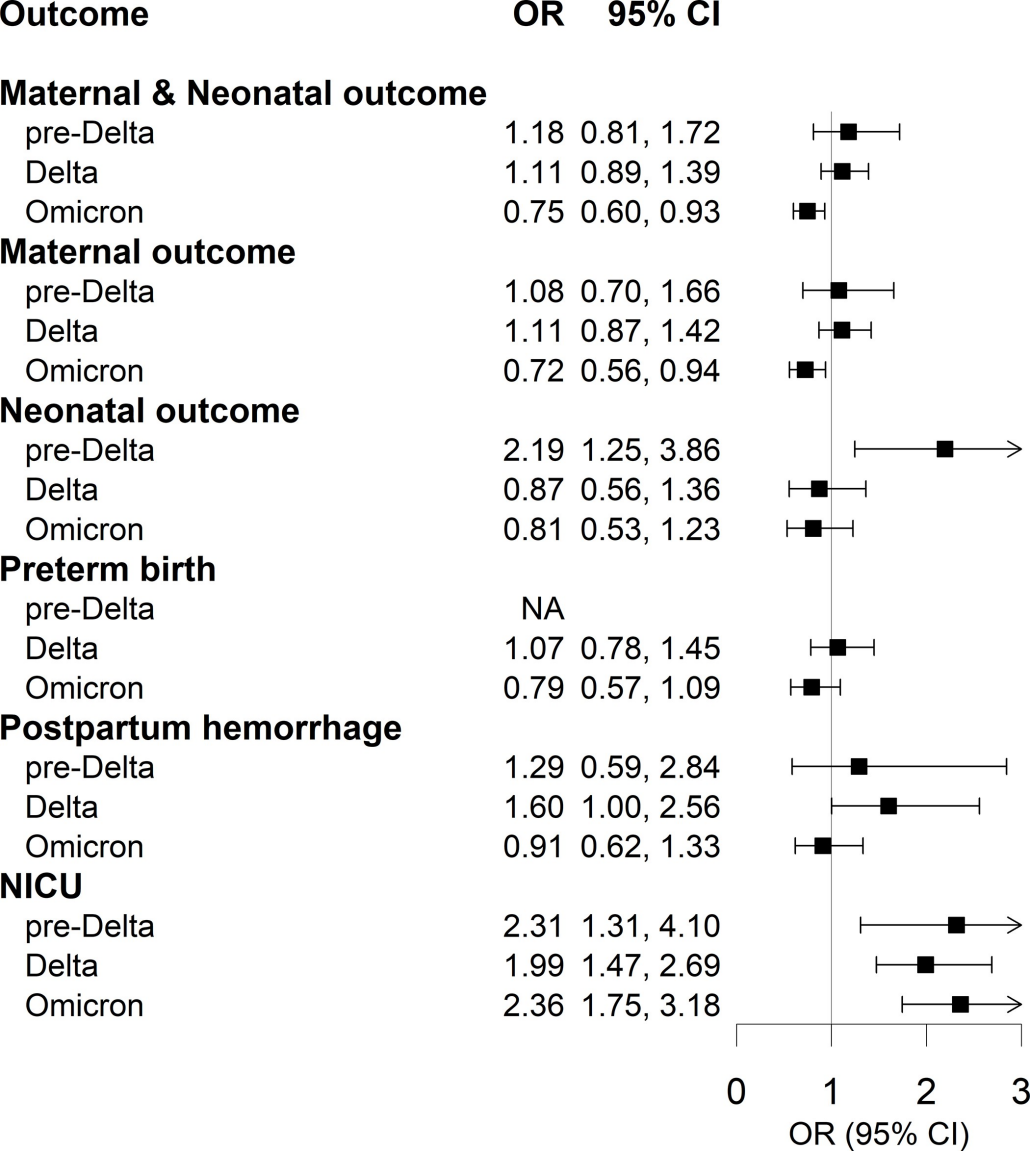
Association of COVID-19 infection during pregnancy with maternal and neonatal adverse outcomes using 1:4 propensity score matching. The arrow indicates when the 95% confidence intervals boundary is beyond the axis limit. In the pre-Delta and Delta periods, the models adjusted for maternal age, sex of child, income, residence area, citizenship, parity, cesarean section, and underlying diseases. The Omicron period, the models adjusted maternal age, sex of child, income, residence area, citizenship, parity, cesarean section, underlying diseases, and vaccination. NA denotes model not applicable. Abbreviations: CI, confidence intervals; OR, odds ratio; NICU: neonatal intensive care unit.

Fig 2 shows the effects of COVID-19 infection during pregnancy on maternal and neonatal outcomes. Overall, the effect of COVID-19 was not observed across all outcomes and periods, except for certain cases (ORs for the pre-Delta period: 1.18, 95% CI: 0.81, 1.72; ORs for the Delta period: 1.11, 95% CI: 0.89, 1.39; ORs for the Omicron period: 0.75, 95% CI: 0.60, 0.93). Moreover, COVID-19 infection increased the risk of PPH in the Delta period (OR: 1.60, 95% CI: 1.00, 2.56) and neonatal outcomes in the pre-Delta period (OR: 2.19, 95% CI: 1.25, 3.86). Even more so, infection with the COVID-19 virus increased the number of admissions to the NICU across all sub-periods (OR of the pre-Delta period: 2.31, 95% CI: 1.31, 4.10; OR of the Delta period: 1.99, 95% CI: 1.47, 2.69; OR of Omicron the period: 2.36, 95% CI: 1.75, 3.18).

**Fig 3:**
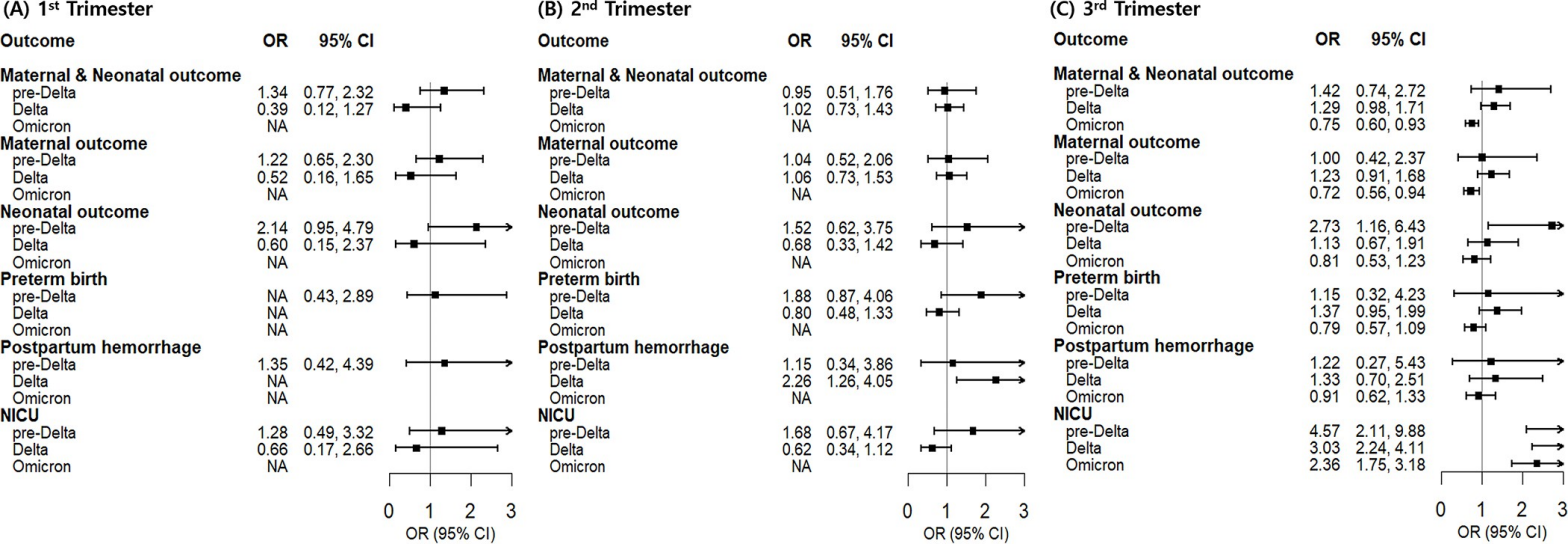
Association of COVID-19 infection in trimester of pregnancy with maternal and neonatal adverse outcomes using 1:4 propensity score matching. The arrow indicates when the 95% confidence intervals boundary is beyond the axis limit. NA denotes model not applicable. Abbreviations: CI, confidence intervals; OR, odds ratio; NICU: neonatal intensive care unit.

Fig 3 displays the trimester-specific results. The effect of COVID-19 on overall maternal and neonatal outcomes was not prominent across each trimester in the pre-Delta period, the Delta period and the Omicron period, respectively. Nevertheless, there were several notable trends that should not be overlooked. For example, PPH in the second trimester of the Delta period (OR: 2.26, 95% CI: 1.26, 4.05), neonatal outcomes in the third trimester of the pre-Delta period (OR: 2.73, 95% CI: 1.16, 6.43), and NICU admissions in the third trimester during the pre-Delta (OR: 4.57, 95% CI: 2.11, 9.88), Delta (OR: 3.03, 95% CI: 2.24, 4.11), and Omicron periods (OR: 2.36, 95% CI: 1.75, 3.18) were all positively associated with COVID-19 infection. On the other hand, during the Omicron period, maternal outcomes in the third trimester were negatively associated with COVID-19 infection.

**Fig 4:**
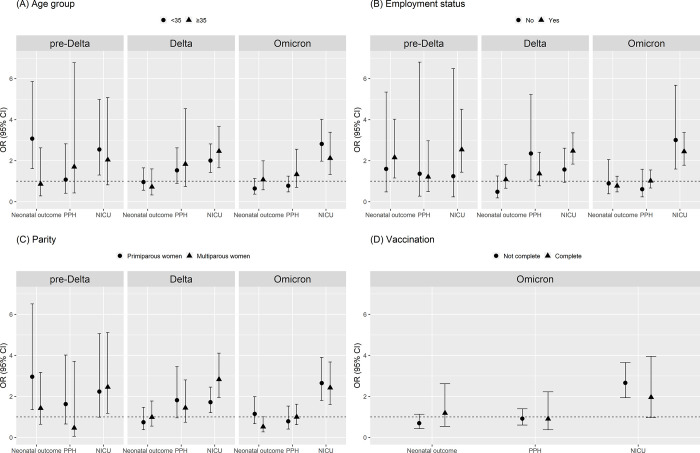
Subgroup analysis of association of COVID-19 infection during pregnancy with adverse outcomes (neonatal outcome, postpartum hemorrhage, and admission to NICU) using 1:4 propensity score matching. Abbreviations: CI, confidence intervals; OR, odds ratio; NICU: neonatal intensive care unit.

Fig 4 presents the results of the subgroup analysis by age groups, employment status, parity, and vaccination status. Unfortunately, we were unable to establish a consistent pattern to determine the risk differences between sub-populations. Based on the point estimate, however, fully vaccinated pregnant women revealed a lower rate of NICU admissions in the Omicron period, compared to mothers who did not have a second vaccine. In addition, pregnant women aged 35 or older generally showed a higher risk of PPH compared to their younger counterparts.

Finally, our sensitivity analysis showed consistent results (S6–S9 Figs) despite different modelling specifications and covariates being considered in the analysis.

## Discussion

To the best of our knowledge, our study is the first NMCC to use data covering the different epidemic periods from the Pre-delta to the early phase of the Omicron variant. In addition, with prospective national cohort data, this study examined the effects of COVID-19 infection on maternal and neonatal outcomes in Korea. Our study indicates that the effect of COVID-19 was sporadically observed in neonatal outcomes and PPH across the sub-periods of COVID-19 variants. Additionally, COVID-19 infections increased admissions to the NICU during the entire variant study periods.

Previous studies have suggested uncertain or mixed results on the effect of COVID-19 infection during pregnancy on the risk of pregnancy-related outcomes (preeclampsia, preterm birth, miscarriage, and perinatal death) [1, 2, 5, 7, 12–16]. In a prior multinational study, a substantial increase in the risk of pregnancy-related complications in women with COVID-19 diagnoses was noted. However, as the authors acknowledged, there were significant differences in the risk of severe maternal morbidity and mortality among developed and less developed nations, as well as the risk of reporting bias [3]. Our study included the entire population of pregnant women in Korea and considered multiple demographic, socioeconomic, and medical histories that could potentially have inherent bias. Thus, with this nationwide cohort, we propose that our study and therefore the results, eliminate any estimate bias and are robust.

Nevertheless, our findings on PPH (positive risk estimates in the second trimester in the Delta sub-periods) are consistent with a previous meta-analysis [6]. Recent literature suggests that COVID-19-induced thrombocytopenia might inhibit physiologic coagulation leading to postpartum bleeding [17–19]. Furthermore, it is also speculated that COVID-19 infection with the Delta variant with relatively strong virulence levels might affect placental development and subclinical placental infection in mid-gestation. This can lead to disturbances to normal hemostasis after delivery, physiologically achieved by complete placental separation and uterine myometrial contraction that directly compresses the avulsed vessels on the implantation site, and can in turn lead to PPH [10, 20]. In the third trimester, particularly towards the end of the pregnancy and close to birth, obstetricians or maternal-fetal-medicine specialists in most of the medical institutions in Korea favored timely Cesarean section to vaginal delivery in COVID-19 infected pregnant women to minimize transmission and as a result, the risk of PPH may be reduced [11, 21, 22]. Additionally, although we included all the delivery cases observed in Korea, our results on PPH were not statistically significant. Because of this, we surmised that the number of infected mothers (2,158 cases) might be insufficient to examine and appropriately determine any causal association between COVID-19 infection and PPH. Therefore, further studies should be implemented.

Despite statistical insignificance, the relative frequency of NICU admissions was higher in the COVID-19 infected group than in the COVID-19 non-infected group during all three periods, in accord with the literature [3, 23]. On December 31, 2020, the Korean Society of Pediatric Infectious Diseases and the Korean Society of Neonatology updated guidelines for the management of newborn infants born to mothers with suspected or confirmed COVID-19 infection, including precautions for healthcare workers, neonatal resuscitation, neonatal isolation and medical care, breastfeeding, testing for COVID-19, and mother/child contact [24]. The national response by government and academia to COVID-19 cases in newborns in Korea was very efficient. This timely policy might have increased the number of NICU admissions but overall it helps to prevent adverse maternal and neonatal outcomes.

Also, from the trimester-specific analyses, there was a positive association between maternal infection in the third trimester and neonatal admission to the NICU. It is hypothesized that the risk of adverse outcomes in neonates who survive and acquire transferred immunity to COVID-19 via the placenta in the first and the second trimesters are at no greater risk than those neonates birthed by non-infected mothers during the same trimesters of pregnancy. Flannery *et al*. stated that the cord blood antibody concentrations correlated with the duration between onset of infection and delivery, and the transfer ratios increased with increasing time [25]. In addition, in Rathberger’s study, a longer period of time between maternal infection and birth appeared to favor the transfer of antibodies to the fetus [26]. This was especially prominent in the third trimester, as on the verge of delivery and birth, the risk of NICU admission was significantly higher in COVID-19 infected groups during the pre-Delta, Delta and Omicron periods. This event is most likely due to insufficient time to obtain immunity and a lack of COVID-19-oriented intensive care as preemptive and preventive measures against the pandemic. During the pre-Delta period, when the systematic medical care for COVID-19 infection was not fully available, the risk of adverse neonatal outcomes, as well as NICU admissions, increased in the COVID-19 infected group.

Based on the large cohort, we investigated the different impact of COVID-19 infection on pregnant women and neonates by sub-populations. Based on the point estimates, we observed several differences. Firstly, during the pre-Delta period, the risk of adverse neonatal outcomes and NICU admission was more pronounced in women of age < 35 years than in women of age ≥35 years; however, this pattern diminished in the Delta and Omicron periods. Secondly, the effect of infection on NICU admission was more prominent in employed mothers in the Delta period, and yet, this trend dissipated in the Omicron period. Nevertheless, across all sub-population results, we could not find any statistically significant differences in risk estimates. Therefore, these results on sub-populations should be interpreted tentatively.

Vaccination during pregnancy is a common way to prevent morbidity from infectious diseases, such as influenza and pertussis. However, COVID-19 vaccines were not explicitly studied in pregnant women and offspring, and so vaccinations for pregnant women were not offered in Korea in October 2021. Although the effect of vaccination was limited in our study, there were few pregnant women who were fully vaccinated during the pre-Delta and the Delta periods. During the Omicron period, the risk of NICU admission was higher in offspring of unvaccinated mothers than offspring of vaccinated mothers. This positive effect of vaccination in pregnant women has been continually recognized in much of the literature [4, 27, 28]. From a study carried out in Israel between January and April 2021, full records of COVID-19 vaccination status and third trimester of pregnancy showed a significant reduction in the risk of composite adverse neonatal outcomes among vaccinated women [29]. In addition, we cautiously speculate that vaccination might play a partial but important role in the negative association between maternal outcomes in the third trimester during the Omicron period and COVID-19 infection.

The main strengths associated with this study are that it provides novel data related to maternal and neonatal outcomes associated with COVID-19 infection during the pre-Delta, Delta and Omicron waves of the pandemic. Moreover, the fact that this nationwide cohort has a relatively large population size enabled this study to include sub-population and trimester-specific analyses as well as a COVID-19 variant-specific analysis. However, only the time of infection was used to indicate the predominant variant due to unavailable variant sequencing data. The Omicron-wave cohort was limited to the third trimester of pregnancy; hence we cannot rule out the possibility of a different course of first or second-trimester pregnancies infected with the Omicron strain. Finally, in this study, we focused on the main maternal outcomes in late gestation; therefore, further studies focusing on early pregnancy losses should be performed in the future.

## Conclusions

To conclude, we found little evidence of the effect of COVID-19 on maternal and neonatal outcomes during the study period. However, we discovered possible evidence that suggests a relationship between COVID-19 infection in the second trimester and PPH, and the association of COVID-19 with increased NICU admissions, which can burden the healthcare system. Therefore, we propose that the timely and successful policies of government and academia in response to COVID-19 infections in newborns in Korea caused an increase in NICU admissions, but nonetheless, prevented adverse maternal and neonatal outcomes simultaneously.

## Supporting information

S1 Text Outcome definitions. (DOCX)

S1 Table Disease-specific diagnostic codes and case definitions. (DOCX)

S2 Table Baseline characteristics for pregnant women before propensity score matching. (DOCX)

S1 Fig Distribution of propensity scores pre-match and post-match propensity score matching. (DOCX)

S2 Fig Scatter plot of propensity score pre-match and post-match propensity score matching. (DOCX)

S3 Fig Love plot of covariate balance pre-match and post-match 1:4 propensity score matching. (DOCX)

S4 Fig The number of first case of COVID-19 infection in pregnancy during study period. The bar plot means the number of first case of COVID-19 infection during pregnancy.
(DOCX)

S5 Fig The numbers of COVID-19 confirmed pregnant women by region in South Korea from January 1, 2020 to March 31, 2022. (DOCX)

S6 Fig Association of COVID-19 infection during pregnancy with maternal and neonatal adverse outcomes using 1:1 propensity score matching. (DOCX)

S7 Fig Association of COVID-19 infection during pregnancy with maternal and neonatal adverse outcomes using stabilized inverse probability of treatment weighting. (DOCX)

S8 Fig Association of COVID-19 infection during pregnancy with maternal and neonatal adverse outcomes using 1:4 propensity score matching after non-Korean exclusion. (DOCX)

S9 Fig Association of COVID-19 infection during pregnancy with maternal and neonatal adverse outcomes using 1:4 propensity score matching when BMI is considered. (DOCX)
