# Supplementary material for: COVID-19, maternal, and neonatal outcomes: National Mother-Child Cohort (NMCC) of K-COV-N cohort in South Korea
Source: PLoS One. 2023 Apr 20;18(4):e0284779. doi: 10.1371/journal.pone.0284779 (PMC10118124; doi:10.1371/journal.pone.0284779)
Supplement: S1 Text — (DOCX) [file pone.0284779.s001.docx]

**S1 Text. Outcome definitions**

The maternal outcomes include hypertensive disorders during pregnancy (HDP), antepartum hemorrhage (APH), PTB, and PPH. Among the maternal outcomes, we examined PTB and PPH as additional outcome variables independently to examine the specific effects on these two outcomes. Meanwhile, the neonatal outcomes include fetal death in utero (FDIU), neonatal respiratory disorders, intraventricular hemorrhage (IVH), hypoxic-ischemic encephalopathy (HIE), neonatal sepsis, neonatal intestinal perforation, retinopathy of prematurity (ROP), and patent ductus arteriosus (PDA). If there was a mother who recorded any of the aforementioned disease or aftereffects during pregnancy or after delivery, then we classified the corresponding outcome variable into ‘Yes’. Theses maternal and neonatal outcomes were defined using the ICD-10 codes as the primary or secondary diagnosis. Detailed disease-specific diagnostic codes and definition criteria are described in S1 Table.
