# Supplementary material for: COVID-19, maternal, and neonatal outcomes: National Mother-Child Cohort (NMCC) of K-COV-N cohort in South Korea
Source: PLoS One. 2023 Apr 20;18(4):e0284779. doi: 10.1371/journal.pone.0284779 (PMC10118124; doi:10.1371/journal.pone.0284779)
Supplement: S1 Table — (DOCX) [file pone.0284779.s002.docx]

**S1 Table. Disease-specific diagnostic codes and case definitions.**

| Outcome | Disease outcomes | Subtypes | ICD-10 | Case definition |
| --- | --- | --- | --- | --- |
| Maternal outcome | Hypertensive disorders in pregnancy (HDP) | Gestational hypertension | O13 | From 20 weeks of pregnancy and posterior one week to the birth of the baby |
|  |  | Preeclampsia | O14, O140-O141, O149 |  |
|  |  | Eclampsia | 015, O150-O152, 0159 |  |
|  |  | HELLP syndrome | O142 |  |
|  |  | Superimposed preeclampsia | O11 |  |
|  | Antepartum hemorrhage (APH) | Threatened abortion | O20 | From 20 weeks of pregnancy and prior to the birth of the baby |
|  |  | Placenta previa | O440-O441 | From 20 weeks of pregnancy and prior to the birth of the baby |
|  |  | Placental abruption | 0450, O458-O459 | From the day before the baby is born to the day the baby is born, |
|  |  | Antepartum hemorrhage, unspecified | 0460, O468-O469 | From 20 weeks of pregnancy and prior to the birth of the baby |
|  |  | Vaginal bleeding | N938-N939 | From 20 weeks of pregnancy and prior to the birth of the baby |
|  | Preterm birth (PTB) |  |  | Less than 37 weeks pregnant |
|  | Postpartum hemorrhage (PPH) | | O720-O721 | Within one week of delivery |
| Neonatal outcome | Fetal death in utero (FDIU) | Abortion (pregnancy loss <20 weeks) | O021, O03 | During pregnancy |
|  |  | Maternal care for intrauterine death | O364 |  |
|  |  | Stillbirth (pregnancy loss >= 20 weeks), singleton | P95, Z3710-Z3713, Z3719 |  |
|  |  | Stillbirth (pregnancy loss >= 20 weeks), twin | Z3730-Z3733, Z3739, Z3740-Z3743, Z3749 |  |
|  |  | Stillbirth (pregnancy loss >= 20 weeks), multiple | Z3760-Z3763, Z3769, Z3770-Z3773, Z3779 |  |
|  | Neonatal respiratory disorders | Respiratory distress syndrome | P220 | Within one week of delivery |
|  |  | Bronchopulmonary dysplasia | P2711, P2712, P2719 |  |
|  |  | Transient tachypnea of newborn | P221 |  |
|  | Interventricular hemorrhage (IVH) |  | P520-P521, P5220-P5221, P523 | Within one week of delivery |
|  | Hypoxic-ischemic encephalopathy  (HIE) |  | P916 | Within one week of delivery |
|  | Neonatal sepsis | Sepsis of new born | P360-P365, P368-P369 | Within one week of delivery |
|  | Neonatal intestinal perforation | Necrotizing enterocolitis | P77 | Within one week of delivery |
|  |  | Perinatal intestinal perforation | P780 | Within one week of delivery |
|  | Retinopathy of prematurity (ROP) |  | H350-H3514, H3519 | Within one week of delivery |
|  | Patent ductus arteriosus (PDA) | Delayed closure of ductus arteriosus | P293, Q250 | Within one week of delivery |
| Admission to Neonatal intensive care unit | Neonatal intensive care unit (NICU) |  |  | Within one week of delivery |
