# Supplementary material for: COVID-19, maternal, and neonatal outcomes: National Mother-Child Cohort (NMCC) of K-COV-N cohort in South Korea
Source: PLoS One. 2023 Apr 20;18(4):e0284779. doi: 10.1371/journal.pone.0284779 (PMC10118124; doi:10.1371/journal.pone.0284779)
Supplement: S2 Table — (DOCX) [file pone.0284779.s003.docx]

**S2 Table. Baseline characteristics for pregnant women before propensity score matching.**

| **Characteristics** | All participants (n=369,887) |
| --- | --- |
| **Continuous variables (Mean** **± SD)** | |
| Maternal age | 32.4 (4.4) |
| GA at delivery | 38.8 (1.6) |
| **Categorical variables (No. (%))** |  |
| **Sex of child** |  |
| Boys | 189,264 (51) |
| Girls | 180,623 (49) |
| **Income-level** |  |
| Unknown | 19,785 (5) |
| Local subscriber : low | 37,019 (10) |
| Local subscriber : mid | 36,280 (10) |
| Local subscriber : high | 10,373 (3) |
| Employee subscriber : low | 107,599 (29) |
| Employee subscriber : mid | 123,201 (33) |
| Employee subscriber : high | 35,630 (10) |
| **Employment status** |  |
| Non-worker | 90,865 (25) |
| Worker | 279,022 (75) |
| **Residence area** |  |
| Region 1 | 190,743 (52) |
| Region 2 | 87,702 (24) |
| Region 3 | 91,442 (25) |
| **Citizenship** |  |
| Domestic | 346,510 (94) |
| Foreign | 23,377 (6) |
| **Parity** |  |
| Primiparous women | 215,001 (58) |
| Multiparous women | 154,886 (42) |
| **Cesarean section** |  |
| No | 163,082 (44) |
| Yes | 206,805 (56) |
| **Underlying disease** |  |
| No | 301,515 (82) |
| Yes | 68,372 (18) |
| **Vaccination** |  |
| Not Complete | 277,277 (75) |
| Complete | 92,610 (25) |

Abbreviations: GA, gestational age
