# Supplementary figures and images for: COVID-19, maternal, and neonatal outcomes: National Mother-Child Cohort (NMCC) of K-COV-N cohort in South Korea
Source: PLoS One. 2023 Apr 20;18(4):e0284779. doi: 10.1371/journal.pone.0284779 (PMC10118124; doi:10.1371/journal.pone.0284779)

**
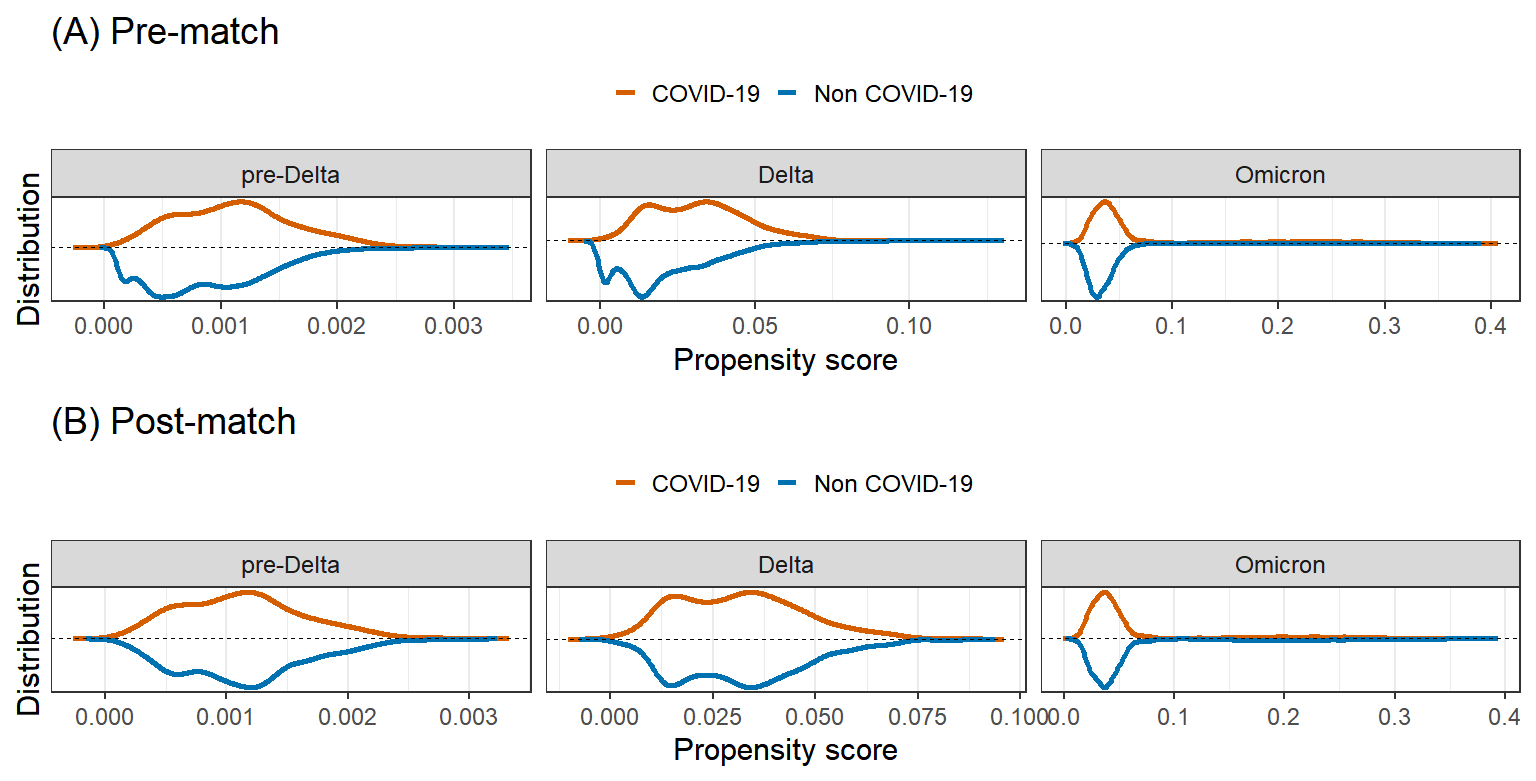
**

**S1 Fig. Distribution of propensity scores pre-match and post-match propensity score matching.**

Supplement: S1 Fig — (DOCX) [file pone.0284779.s004.docx]

**
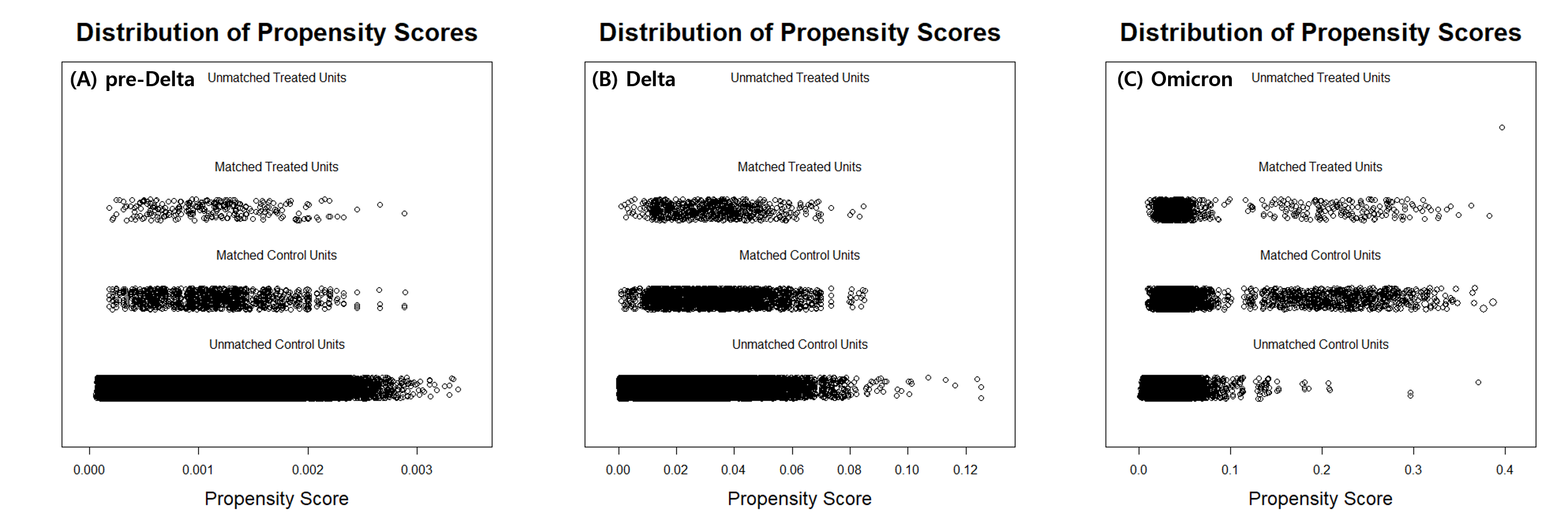
**

**S2 Fig. Scatter plot of propensity score pre-match and post-match propensity score matching.**

Supplement: S2 Fig — (DOCX) [file pone.0284779.s005.docx]

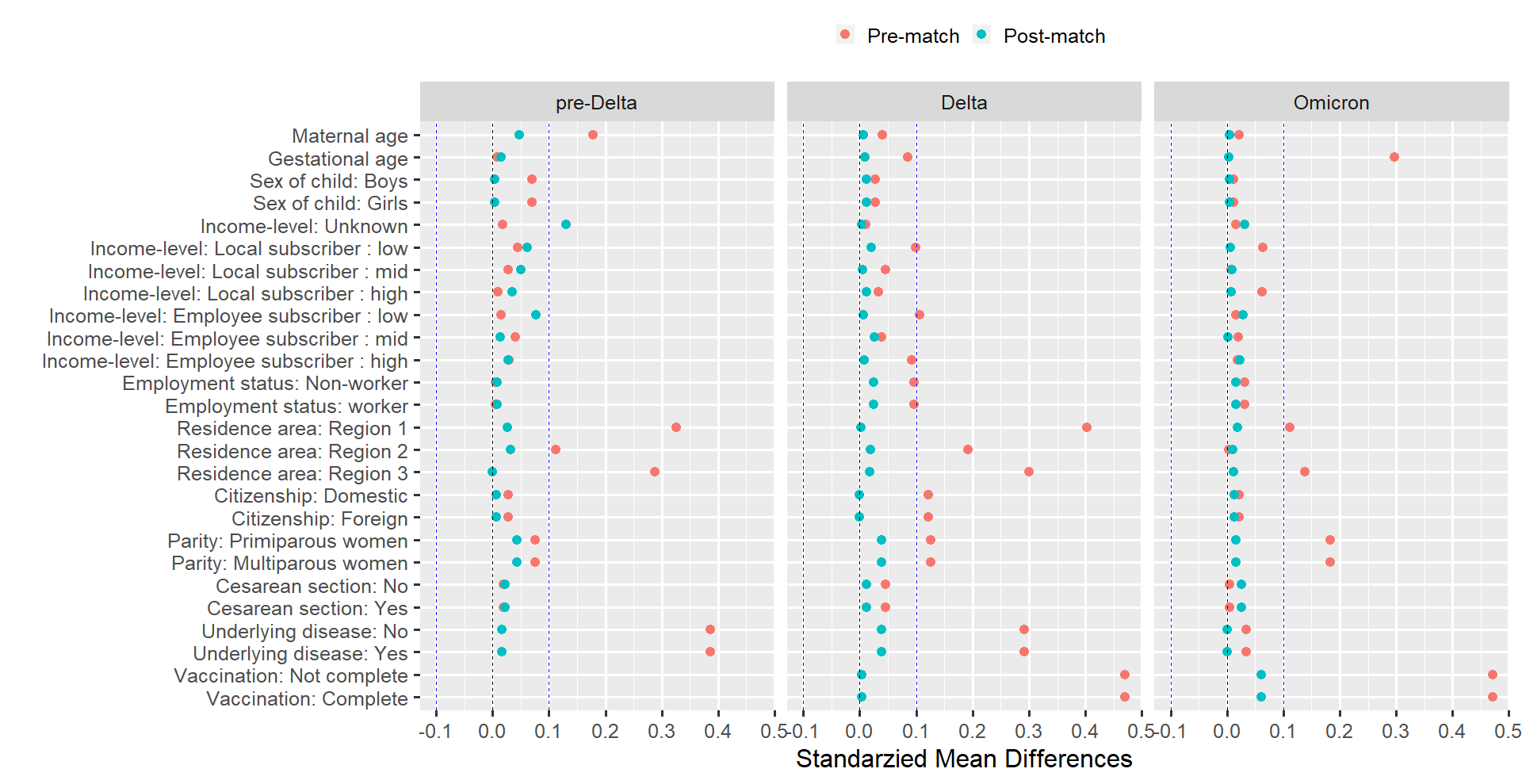


**S3 Fig. Love plot of covariate balance pre-match and post-match 1:4 propensity score matching.**

Supplement: S3 Fig — (DOCX) [file pone.0284779.s006.docx]
