# Supplementary material for: COVID-19, maternal, and neonatal outcomes: National Mother-Child Cohort (NMCC) of K-COV-N cohort in South Korea
Source: PLoS One. 2023 Apr 20;18(4):e0284779. doi: 10.1371/journal.pone.0284779 (PMC10118124; doi:10.1371/journal.pone.0284779)
Supplement: S4 Fig — The bar plot means the number of first case of COVID-19 infection during pregnancy. (DOCX) [file pone.0284779.s007.docx]

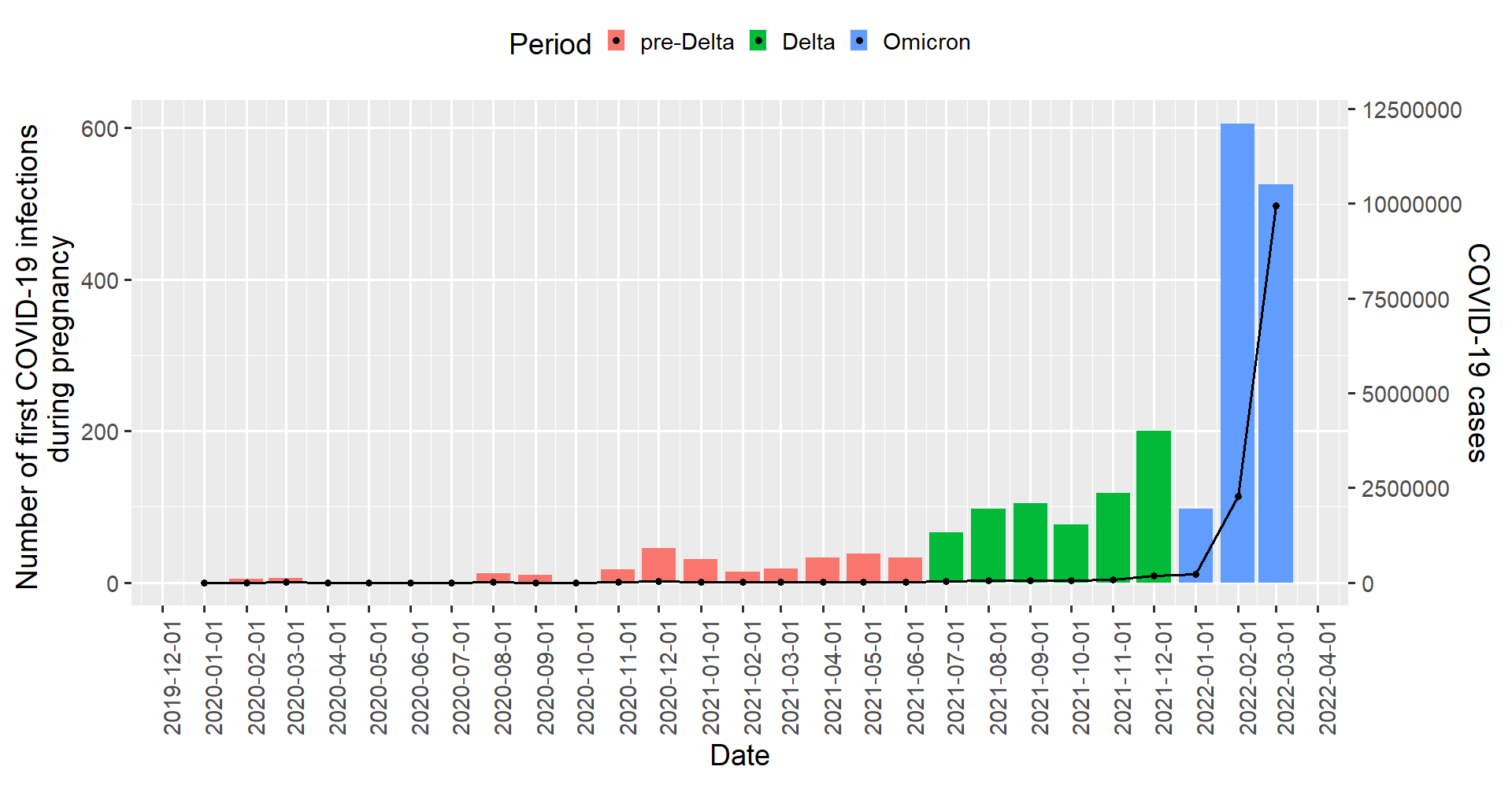


**S4 Fig. The number of first case of COVID-19 infection in pregnancy during study period. The bar plot means the number of first case of COVID-19 infection during pregnancy.**

The auxiliary axis means the number of COVID-19 infections during the study period.

pre-Delta (January 2020-June 2021), Delta (July 2021-December 2021), and Omicron (January 2022-March 2022) periods.
