# Supplementary material for: COVID-19, maternal, and neonatal outcomes: National Mother-Child Cohort (NMCC) of K-COV-N cohort in South Korea
Source: PLoS One. 2023 Apr 20;18(4):e0284779. doi: 10.1371/journal.pone.0284779 (PMC10118124; doi:10.1371/journal.pone.0284779)
Supplement: S5 Fig — (DOCX) [file pone.0284779.s008.docx]

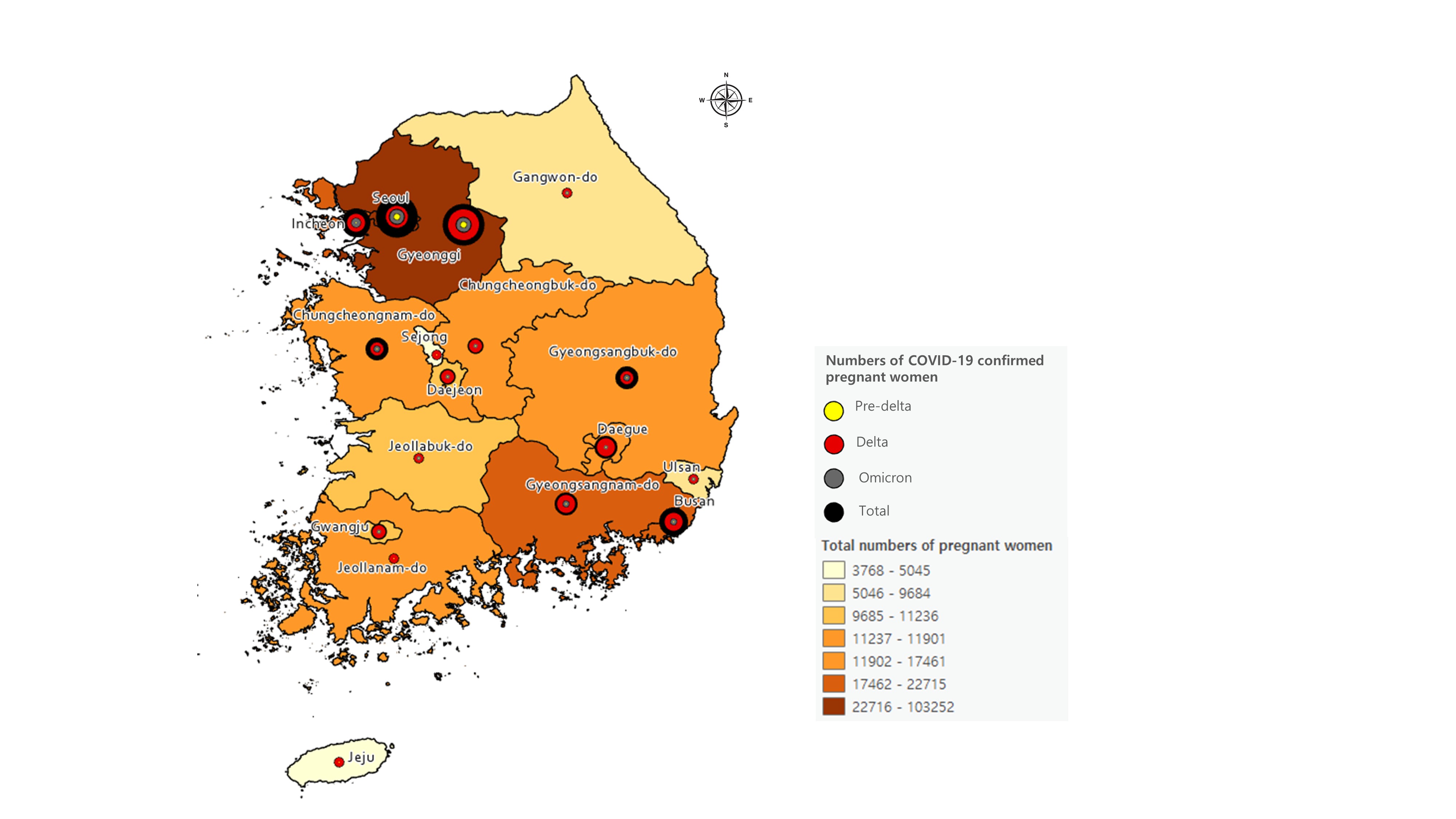


**S5 Fig. The numbers of COVID-19 confirmed pregnant women by region in South Korea from January 1, 2020 to March 31, 2022.**

pre-Delta (January 2020-June 2021), Delta (July 2021-December 2021), and Omicron (January 2022-March 2022) periods.
