# Supplementary material for: COVID-19, maternal, and neonatal outcomes: National Mother-Child Cohort (NMCC) of K-COV-N cohort in South Korea
Source: PLoS One. 2023 Apr 20;18(4):e0284779. doi: 10.1371/journal.pone.0284779 (PMC10118124; doi:10.1371/journal.pone.0284779)
Supplement: S7 Fig — (DOCX) [file pone.0284779.s010.docx]

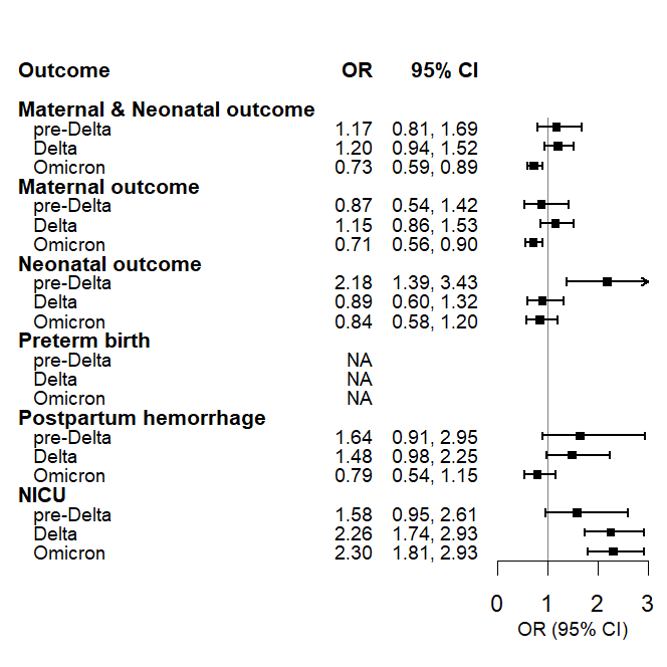


**S7 Fig. Association of COVID-19 infection during pregnancy with maternal and neonatal adverse outcomes using stabilized inverse probability of treatment weighting.**

The arrow is when the 95% confidence intervals boundary is beyond the axis limit. In pre Delta and Delta period, the models adjusted maternal age, sex of child, income, residence area, citizenship, parity, cesarean section, and underlying diseases. Omicron period, the models adjusted maternal age, sex of child, income-level, employment status, residence area, citizenship, parity, cesarean section, underlying diseases, and vaccination. NA denotes model not applicable.

Abbreviations: CI, confidence intervals; OR, odds ratio; NICU: neonatal intensive care unit
