# Supplementary material for: COVID-19, maternal, and neonatal outcomes: National Mother-Child Cohort (NMCC) of K-COV-N cohort in South Korea
Source: PLoS One. 2023 Apr 20;18(4):e0284779. doi: 10.1371/journal.pone.0284779 (PMC10118124; doi:10.1371/journal.pone.0284779)
Supplement: S8 Fig — (DOCX) [file pone.0284779.s011.docx]

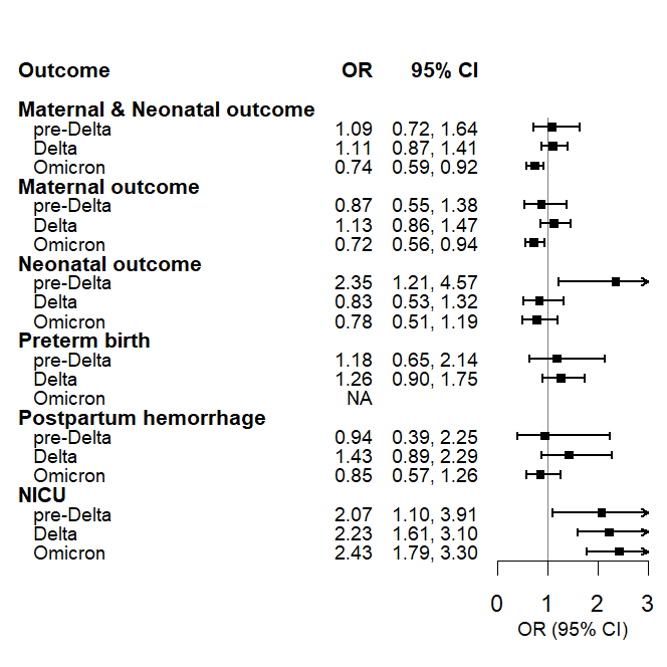


**S8 Fig. Association of COVID-19 infection during pregnancy with maternal and neonatal adverse outcomes using 1:4 propensity score matching after non-Korean exclusion.**

The arrow is when the 95% confidence intervals boundary is beyond the axis limit. In pre Delta and Delta period, the models adjusted maternal age, sex of child, income-level, employment status, residence area, citizenship, parity, cesarean section, and underlying diseases. Omicron period, the models adjusted maternal age, sex of child, income-level, employment status, residence area, citizenship, parity, cesarean section, underlying diseases, and vaccination. NA denotes model not applicable.

Abbreviations: CI, confidence intervals; OR, odds ratio; NICU: neonatal intensive care unit
